# Novel Insight Into Long-Term Risk of Major Adverse Cardiovascular and Cerebrovascular Events Following Lower Extremity Arteriosclerosis Obliterans

**DOI:** 10.3389/fcvm.2022.853583

**Published:** 2022-04-04

**Authors:** Ji Sun, Qiang Deng, Jun Wang, Shoupeng Duan, Huaqiang Chen, Huixin Zhou, Zhen Zhou, Fu Yu, Fuding Guo, Chengzhe Liu, Saiting Xu, Lingpeng Song, Yijun Wang, Hui Feng, Lilei Yu

**Affiliations:** ^1^Department of Cardiology, Renmin Hospital of Wuhan University, Cardiac Autonomic Nervous System Research Centre of Wuhan University, Cardiovascular Research Institute, Wuhan University, Hubei Key Laboratory of Cardiology, Wuhan, China; ^2^Information Center, Renmin Hospital of Wuhan University, Wuhan, China

**Keywords:** lower extremity arteriosclerosis obliterans, major cardiovascular and cerebrovascular adverse events, gender, panvascular disease, coronary artery disease

## Abstract

**Background:**

Patients with lower extremity arteriosclerosis obliterans (LEASO) are more likely to appear to be associated with adverse cardiovascular outcomes. Currently, few studies have reported the sex-specific characteristics and risk of major cardiovascular and cerebrovascular adverse events (MACCEs) in LEASO. Our study was conducted to determine the characteristics and contributions of LEASO to MACCEs in males and females.

**Methods:**

We conducted a single-center retrospective study of consecutively enrolled patients with first-diagnosed LEASO at Renmin Hospital of Wuhan University from November 2017 to November 2019. The ratio of patients between the LEASO and control groups was 1 to 1 and based on age, sex, comorbid diabetes mellitus and hypertension, current smoking and medications. The occurrence of MACCEs was used as the primary endpoint of this observational study.

**Results:**

A LEASO group (*n* = 430) and control group (*n* = 430) were enrolled in this study. A total of 183 patients experienced MACCEs during an average of 38.83 ± 14.28 months of follow-up. Multivariate Cox regression analysis indicated that LEASO was an independent predictor of the occurrence of MACCEs in all patients (HR: 2.448, 95% CI: 1.730–3.464, *P* < 0.001). Subgroup analysis by sex subgroup was conducted for sex, and LEASO was also an independent predictor of the occurrence of MACCEs in both male cases (HR: 2.919, 95% CI: 1.776–4.797, *P* < 0.001) and female cases (HR: 1.788, 95% CI: 1.110–2.880, *P* = 0.017). Moreover, Kaplan–Meier analysis indicated no significant difference in event-free survival between patients of different sexes with LEASO (χ^2^ = 0.742, *P* = 0.389).

**Conclusion:**

LEASO tended to a useful risk stratified indicator for MACCEs in both male and female patients in our study. Notably, attention should be given to patients with LEASO who should undergo comprehensive cardiovascular evaluation and intervention, even if there is a lack of traditional cardiovascular risk factors.

## Graphical Abstract

Central illustration: lower extremity arteriosclerosis obliterans as a prognostic factor for the occurrence of major cardiovascular and cerebrovascular adverse events.

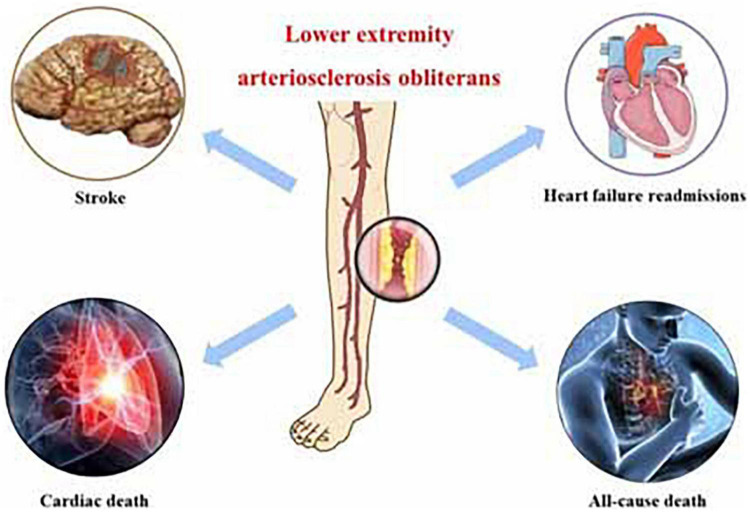

## Introduction

Lower extremity arteriosclerosis obliterans (LEASO), the main and most common type of lower extremity peripheral arterial disease (PAD), tends to increase with age (1, 2). Notably, accumulating evidence suggests that people suffering from PAD are at higher risk of other health risks, including cardiovascular death, stroke, heart failure, and myocardial infarction (MI) (3, 4). Furthermore, patients with lower extremity PAD maintained higher cardiovascular (CV) mortality than MI patients due to less intensive treatment, which is closely associated with heart failure hospitalization, ischemic stroke and CV death (5, 6). Notably, LEASO shares cardiovascular risk factors in common with coronary heart disease (CHD) and may also have a similar pathophysiologic basis. Therefore, it is significant for patients with LEASO to carry out comprehensive cardiovascular follow-up examinations and medical prevention.

It is well known that sex is a substantial unchangeable cardiovascular risk factor, and men and women differ in terms of characteristics and management of coronary artery disease (CAD) (7, 8). A previous study showed that females with acute ischemic stroke who received parallel in-hospital care had more vascular risk factors and were more likely to be discharged with disability (9). In addition, female patients with CAD carry a higher risk of heart failure, ischemic stroke and all-cause mortality than male patients with CAD (10). Nevertheless, men with AF reported better overall health-related quality of life (11). Moreover, males and females also differ with regard to key features of other cardiovascular diseases (12–14). Although lower extremity PAD is a component of systemic atherosclerosis and carries a dramatically heightened risk of cardiovascular morbidity and mortality, sex differences were not included in further analyses (3, 15). Moreover, existing data are limited to correcting for a possible confounder, including routine laboratory results and drug treatments. Therefore, in this study, we aimed to evaluate whether LEASO could serve as an independent predictor of major cardiovascular and cerebrovascular adverse events (MACCEs) and determine whether these quantitative assessments provide parallel prognostic intelligence in males and females.

## Materials and Methods

### Study Population

A single-center retrospective cohort study was launched at the Renmin Hospital of Wuhan University. In total, 430 consecutive patients with a first diagnosis of LEASO and without a history of LEASO or CAD who received optimal clinical intervention from November 2017 to November 2019 were enrolled in our retrospective study. Patients suffering from a history of LEASO, prior CAD, malignancy, severe renal insufficiency (eGFR < 30 ml/min), severe liver disease, stroke, and severe lung disease were not recruited for the study. Individuals without LEASO who underwent coronary angiogram to rule out CAD in the same period were included in the control group (*n* = 430), which was matched with the LEASO group at a 1-to-1 ratio, according to age, sex, diabetes, hypertension, current smoking status, and medications.

### Data Collection

Venous blood taken from all patients on the day of admission was sent to the Department of Clinical Laboratory of Renmin Hospital of Wuhan University to measure the parameters of routine blood examination and biochemistry, such as white blood cell count (WBC), lymphocyte cell count, neutrophil cell count, platelet count, neutrophil-to-lymphocyte ratio (NLR), platelet-to-lymphocyte ratio (PLR), uric acid (UA), glucose, total cholesterol (TC), total triglycerides (TG), high-density lipoprotein cholesterol (HDL-c), low-density lipoprotein cholesterol (LDL-C), apolipoprotein A1 (Apo A1), apolipoprotein B (Apo B), lipoprotein a, hypersensitive C-reactive protein (hs-CRP), total bilirubin (TBil), direct bilirubin (DBil), fibrinogen, and D-dimers.

### Follow-Up and End-Point

After discharge, all the patients were followed-up by telephone or outpatient visits, and the mean follow-up time was approximately 38.83 months. The observed outcome of this study was identified as the occurrence of MACCEs together with (a) all-cause mortality, (b) cardiac mortality, (c) acute coronary syndromes, (d) stroke, (e) admission to the hospital necessitated by heart failure, (f) admission to the hospital necessitated by atrial fibrillation, and (g) revascularization.

### Propensity Score Matching

Potential confounding factors were controlled by matching the covariates of the LEASO groups and controls group as many as possible based on the propensity scores calculated, implementing logistic multiple regression analysis after applying propensity scores matching (PSM), as recommended in the literature (16). Final covariates were age, sex, diabetes, hypertension, current smoking status, and medications according to the results of the pre-survey. Propensity score analysis with 1-to-1 ratio matching and the nearest neighbor matching method was applied to ensure well-balanced features between the LEASO groups and controls group. The propensity score with a standard caliper width of 0.2.

### Statistical Methods

The mean and standard deviation (SD) or median and interquartile range (IQR) were applied to our results to represent continuous variables, whereas percentage was used to represent categorical variables. For data processing methods, *t* tests were used for continuous variables, and chi-square (χ2) tests were used for categorical variables. The Kaplan–Meier survival method was applied to identify prognostic factors for the occurrence of MACCEs. The Kaplan–Meier survival curves were compared using the logrank test. Univariate analysis was performed first, and the significant variables were included in a subsequent multivariate Cox analysis. Comparisons were performed to analyze whether adding LEASO to the traditional cardiovascular risk factors, including gender, age, hypertension, diabetes, current smoking, current drinking, for MACCEs could improve the predictive ability of the models. The addition of LEASO to the existing models 1 with the traditional cardiovascular risk was evaluated with the predicted probabilities of MACCEs, using increase in the area under the receiver operating characteristic curve (AUC), sensitivity, specificity and C-index and Youden index. A statistically significant difference was denoted when the *P* value was < 0.05. SPSS 23.0 (SPSS, Inc., Chicago, IL, United States) was applied for all analyses.

## Results

### Patient Characteristics of the Lower Extremity Arteriosclerosis Obliterans Group and Control Group

A total of 860 patients were identified, of whom 430 were diagnosed with LEASO. Four-hundred thirty patients composed a control group, and their clinical characteristics are shown in Table 1. The data showed that white blood cell count (*P* < 0.001), neutrophil cell count (*P* < 0.001), NLR (*P* < 0.001), PLR (*P* = 0.002), glucose (*P* < 0.001), TG (*P* = 0.001), TC (*P* < 0.001), LDL-C (*P* = 0.002), Apo B (*P* = 0.27), Lp (a) (*P* = 0.017), fibrinogen (*P* < 0.001), D-dimer (*P* < 0.001), and DBil (*P* < 0.001) tended to be higher in LEASO patients than in the control patients. In addition, patients with LEASO remained more likely to have lower lymphocyte counts (*P* < 0.001), HDL-C levels (*P* = 0.001), and Apo A1 levels (*P* < 0.001) than did the control group patients.

**TABLE 1:** Characteristics of LEASO group and control group.

	Control (*n* = 430)	LEASO (*n* = 430)	*t*/*Z*/χ 2	*p*
Gender (male)	205 (47.7)	223 (51.9)	1.507	0.220
Age (years)	65.75 ± 7.98	66.55 ± 11.54	1.182	0.237
Hypertension (%)	203 (47.2)	216 (50.2)	0.787	0.375
Duration of Hypertension (years)	10.00 (5.00, 15.00)	10.00 (6.00, 15.00)	1.179	0.238
Diabetes (%)	66 (15.3)	87 (20.2)	3.506	0.061
Duration of Diabetes (years)	9.00 (3.00, 10.25)	10.00 (5.00, 12.00)	1.765	0.078
Current Smoking (%)	87 (20.2)	106 (24.7)	2.412	0.120
Duration of smoking (years)	30.00 (20.00, 35.00)	30.00 (20.00, 40.00)	0.255	0.799
Current smoking cigarettes (per day)	20.00 (10.00, 20.00)	20.00 (10.00, 20.00)	0.956	0.339
Current Drinking (%)	44 (10.2)	56 (13.0)	1.629	0.202
WBC (×10^9^/L)	6.09 ± 1.72	7.74 ± 3.72	8.375	<0.001
Neutrophil (×10^9^/L)	3.82 ± 1.63	5.34 ± 3.47	8.218	<0.001
Lymphocyte (×10^9^/L)	1.72 ± 0.56	1.54 ± 0.59	4.653	<0.001
NLR	2.04 (1.51, 2.83)	2.82 (1.88, 4.84)	8.348	<0.001
PLT (×10^9^/L)	212.60 ± 58.21	214.32 ± 84.65	0.346	0.729
PLR	124.81 (101.30, 156.37)	136.92 (98.58, 188.62)	3.060	0.002
Creatinine (μmol/L)	73.14 ± 29.43	71.40 ± 12.19	1.131	0.259
UA (μmol/L)	354.17 ± 99.39	368.37 ± 116.86	1.920	0.055
Glucose (mmol/L)	5.61 ± 1.70	6.21 ± 2.57	3.993	<0.001
TG (mmol/L)	1.13 (0.87, 1.38)	1.20 (0.90, 1.67)	3.433	0.001
TC (mmol/L)	3.74 ± 0.59	4.03 ± 1.04	4.990	<0.001
HDL-C (mmol/L)	1.15 ± 0.31	1.08 ± 0.31	3.454	0.001
LDL-C (mmol/L)	2.12 ± 0.56	2.27 ± 0.87	3.047	0.002
Apo A1 (g/L)	1.36 ± 0.22	1.27 ± 0.22	6.307	<0.001
Apo B (g/L)	0.73 ± 0.17	0.76 ± 0.23	2.217	0.027
Lp (a) (g/L)	145.50 (68.00, 305.38)	189.00 (80.75, 401.50)	2.379	0.017
hs-CRP (mg/L)	1.89 (0.32, 8.98)	1.52 (0.58, 5.98)	1.359	0.174
Fibrinogen (g/L)	2.90 ± 0.74	3.62 ± 1.36	9.551	<0.001
D-dimer (ug/ml)	0.24 (0.17, 0.41)	0.73 (0.38, 1.56)	14.976	<0.001
TBil (μmol/L)	11.63 (9.18, 15.60)	12.00 (8.88, 15.70)	0.429	0.668
DBil (μmol/L)	3.40 (2.70, 4.40)	4.00 (3.08, 5.80)	5.577	<0.001
**Medications**				
Aspirin (%)	284 (66.0)	262 (60.9)	2.428	0.119
Statins (%)	291 (67.7)	277 (64.4)	1.016	0.313
β-blocker (%)	113 (26.3)	106 (24.7)	0.300	0.584
ACEI/ARB (%)	48 (11.2)	45 (10.5)	0.109	0.742
CCB (%)	109 (25.3)	86 (20.0)	3.508	0.061

*WBC, white blood cell count; NLR, neutrophil-to-lymphocyte ratio; PLT, platelet count; PLR, platelet-to-lymphocyte ratio; UA, uric acid; TG, triglycerides; TC, total cholesterol; HDL-C, high-density lipoprotein cholesterol; LDL-C, low-density lipoprotein cholesterol; Apo A1, Apolipoprotein A1; Apo B, Apolipoprotein B; Lp (a), lipoprotein a; hs-CRP, hypersensitive C-reactive protein; TBil, total bilirubin; DBil, direct bilirubin; ACEI, angiotensin converting enzyme inhibitor; ARB, angiotensin receptor blockers; CCB, calcium ion channel blockers; LEASO, lower extremity arteriosclerosis obliterans.*

### Predictors of Patients’ Clinical Outcomes

**FIGURE 1:**
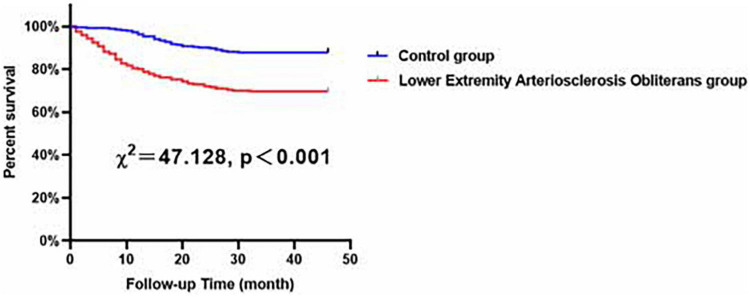
Major adverse cardiovascular and cerebrovascular events (MACCEs)-free survival rates for patients with LEASO group and control group during the follow-up period.

In our study, patients were followed-up for 38.83 ± 14.28 months. The clinical outcomes of all patients are presented in Table 2, and a total of 183 patients suffered from MACCEs during the follow-up period. Our study showed that, compared to the control group patients, patients subjected to LEASO tended to have a higher incidence of MACCEs (*P* < 0.001), all-cause death (*P* < 0.001), cardiac death (*P* < 0.001), stroke (*P* = 0.002) and admission to the hospital necessitated by heart failure (*P* < 0.001). According to Kaplan–Meier analysis, an obvious difference could be found in the incidence of MACCEs between the LEASO patients and controls (χ^2^ = 47.128, *p* < 0.001), and the incidence of MACCEs in the LEASO group was higher than that in the control group, as shown in Figure 1.

**TABLE 2:** Incidence of MACCEs of LEASO group and control group.

	Control (*n* = 430)	LEASO (*n* = 430)	χ2	*p*
MACCEs	52 (12.1)	131 (30.5)	43.322	<0.001
All-cause death	1 (0.2)	75 (17.4)	79.037	<0.001
Cardiac death	1 (0.2)	39 (9.1)	37.861	<0.001
Revascularization	4 (0.9)	10 (2.3)	2.614	0.106
Stroke	17 (4.0)	40 (9.3)	9.939	0.002
Acute coronary syndromes	37 (8.6)	31 (7.2)	0.575	0.448
Admission to the hospital necessitated by Atrial fibrillation	13 (3.0)	10 (2.3)	0.402	0.526
Admission to the hospital necessitated by Heart failure	5 (1.2)	37 (8.6)	25.633	<0.001

According to univariate Cox analysis, hypertension, diabetes, LEASO, WBC, neutrophil, lymphocyte, NLR, PLR, UA, Apo A1, fibrinogen, D-dimers, and the application of aspirin and β-blockers were predictors of MACCEs, as shown in Table 3. Multivariate Cox analysis was then applied to identify independent influencing factors that predict MACCEs in patients. The results showed that hypertension (HR: 1.795, 95% CI: 1.320–2.440, *P* < 0.001), NLR (HR: 1.109, 95% CI: 1.057–1.163, *P* < 0.001), and LEASO (HR: 2.448, 95% CI: 1.730–3.464, *P* < 0.001) were independent risk factors for MACCEs, as presented in Table 3 (Central illustration). Moreover, the application of aspirin (HR: 0.608, 95% CI: 0.450–0.821, *P* = 0.001) or β-blockers (HR: 0.423, 95% CI: 0.280–0.638, *P* < 0.001) remained a protective factor for MACCEs, as shown in Table 3.

**TABLE 3:** Predictors of the occurrence of MACCEs in LEASO patients: results of univariate and multivariate Cox-regression analyses.

Indicators	Univariate			Multivariate		
	HR	95%CI	*p*	HR	95%CI	*p*
Gender (female)	1.134	0.848–1.515	0.397			
Age (years)	0.999	0.984–1.014	0.891			
Hypertension (%)	1.759	1.307–2.368	<0.001	1.795	1.320–2.440	<0.001
Diabetes (%)	1.478	1.051–2.081	0.025	1.242	0.872–1.771	0.230
Current Smoking (%)	1.282	0.922–1.781	0.140			
Current Drinking (%)	0.921	0.579–1.466	0.730			
LEASO (%)	2.914	2.113–4.019	<0.001	2.448	1.730–3.464	<0.001
WBC (×10^9^/L)	1.086	1.049–1.124	<0.001	0.971	0.918–1.028	0.308
Neutrophil (×10^9^/L)	1.106	1.067–1.146	<0.001			
Lymphocyte (×10^9^/L)	0.643	0.491–0.841	0.001			
NLR	1.104	1.075–1.134	<0.001	1.109	1.057–1.163	<0.001
PLT (×109/L)	0.998	0.996–1.000	0.098			
PLR	1.002	1.001–1.004	0.006	0.998	0.996–1.001	0.198
Creatinine (μmol/L)	1.002	0.996–1.008	0.464			
UA (μmol/L)	1.001	1.000–1.003	0.046	1.000	0.999–1.002	0.449
Glucose (mmol/L)	1.043	0.983–1.108	0.165			
TG (mmol/L)	0.992	0.812–1.212	0.940			
TC (mmol/L)	1.042	0.880–1.233	0.635			
HDL-C (mmol/L)	0.654	0.400–1.071	0.091			
LDL-C (mmol/L)	0.997	0.817–1.217	0.976			
Apo A1 (g/L)	0.493	0.259–0.939	0.032	0.882	0.450–1.731	0.716
Apo B (g/L)	1.109	0.532–2.310	0.782			
Lp (a) (g/L)	1.001	0.999–1.001	0.983			
hs-CRP (mg/L)	1.001	0.994–1.007	0.800			
Fibrinogen (g/L)	1.231	1.112–1.362	<0.001	1.057	0.923–1.210	0.424
D-dimer (ug/ml)	1.038	1.009–1.069	0.010	0.982	0.931–1.035	0.502
TBil (μmol/L)	1.005	0.987–1.023	0.590			
DBil (μmol/L)	1.023	0.995–1.052	0.109			
**Medications**						
Aspirin (%)	0.677	0.506–0.906	0.009	0.608	0.450–0.821	0.001
Statins (%)	0.928	0.685–1.258	0.630			
β-blocker (%)	0.472	0.314–0.711	<0.001	0.423	0.280–0.638	<0.001
ACEI/ARB (%)	1.236	0.798–1.913	0.342			
CCB (%)	1.236	0.889–1.718	0.207			

*HR, hazard ratio; CI, confidence interval.*

**FIGURE 2:**
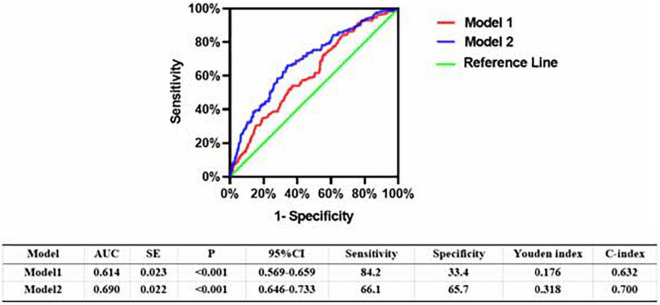
Receiver operating character analysis for the predictive efficacy of variables for MACCEs (Model 1, Gender + Age + Hypertension + Diabetes + current smoking + current drinking; Model 2, Model 1 + LEASO).

In the multivariable analysis model, when added to clinical risk factors, LEASO increased the discriminatory indices (Figure 2). Distribution of predicted risks for MACCEs from model 1 based on age, sex, hypertension, diabetes, current smoking, and current drinking (AUC: 0.614; C-index: 0.632; Youden index: 0.176; sensitivity: 84.2%; specificity: 33.4%; *P* < 0.001). For the predictability of MACCEs, the positive Youden index of the combined LEASO increased in model 2 (AUC: 0.690; C-index: 0.700; Youden index: 0.318; sensitivity: 66.1%; specificity: 65.7%; *P* < 0.001). This suggested that incorporating LEASO enhanced the ability to predict accurately the MACCEs compared with Model 1, which included traditional cardiovascular risk factors only (AUC: 0.690 versus 0.614; C-index: 0.700 versus 0.632).

### Predictors of Clinical Outcomes in Male Patients

**FIGURE 3:**
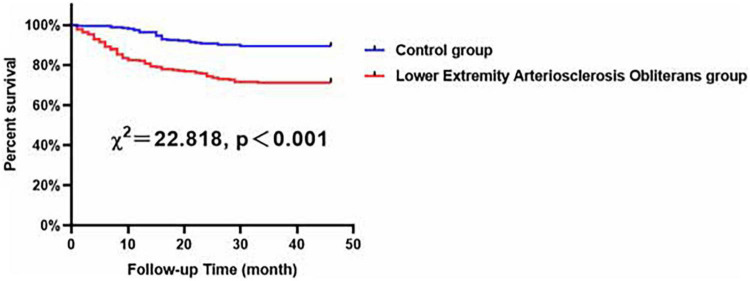
Major adverse cardiovascular and cerebrovascular events-free survival rates for male patients with LEASO group and control group during the follow-up period.

According to Kaplan–Meier analysis, in all of the evaluated male patients, compared with the control patients, the patients with LEASO seemed to maintain lower MACCE-free survival rates (χ^2^ = 22.818, *P* < 0.001, Figure 3).

According to univariable Cox analysis, diabetes, current smoking, LEASO, NLR, and HDL-C (all *P* < 0.05) were predictors of MACCEs in all of the evaluated male patients (Table 4). Moreover, multivariate Cox analysis indicated that diabetes (HR: 1.725, 95% CI: 1.068–2.787, *P* = 0.026), current smoking (HR: 1.734, 95% CI: 1.133–2.652, *P* = 0.011), LEASO (HR: 2.919, 95% CI: 1.776–4.797, *P* < 0.001), and HDL-C (HR: 0.269, 95% CI: 0.117–0.620, *P* = 0.002) were independent influencing factors for MACCEs in all of the evaluated male patients (Table 4).

**TABLE 4:** Predictors of the occurrence of MACCEs in male patients: results of univariate and multivariate Cox-regression analyses.

Indicators	Univariate			Multivariate		
	HR	95%CI	*p*	HR	95%CI	*p*
Age (years)	1.006	0.984–1.028	0.606			
Hypertension (%)	1.397	0.914–2.136	0.123			
Diabetes (%)	1.918	1.190–3.092	0.008	1.725	1.068–2.787	0.026
Current Smoking (%)	1.670	1.093–2.551	0.018	1.734	1.133–2.652	0.011
Current Drinking (%)	0.989	0.600–1.631	0.966			
LEASO (%)	3.058	1.884–4.966	<0.001	2.919	1.776–4.797	<0.001
WBC (×10^9^/L)	1.050	0.992–1.112	0.094			
Neutrophil (×10^9^/L)	1.054	0.991–1.120	0.095			
Lymphocyte (×10^9^/L)	0.829	0.562–1.222	0.343			
NLR	1.069	1.016–1.126	0.011	1.044	0.985–1.106	0.148
PLT (×10^9^/L)	0.998	0.995–1.001	0.260			
PLR	1.001	0.998–1.004	0.471			
Creatinine (mmol/L)	1.006	0.998–1.014	0.162			
UA (mmol/L)	1.001	0.999–1.002	0.671			
Glucose (mmol/L)	0.939	0.845–1.044	0.242			
TG (mmol/L)	1.106	0.826–1.482	0.498			
TC (mmol/L)	1.097	0.853–1.411	0.471			
LDL-C (mmol/L)	0.920	0.684–1.239	0.585			
HDL-C (mmol/L)	0.242	0.101–0.584	0.002	0.269	0.117–0.620	0.002
Apo A1 (g/L)	0.933	0.341–2.548	0.892			
Apo B (g/L)	0.569	0.170–1.897	0.358			
Lp (a) (g/L)	1.001	0.999–1.001	0.390			
hs-CRP (mg/L)	0.999	0.988–1.009	0.803			
Fibrinogen (g/L)	1.124	0.968–1.304	0.124			
D-dimer (ug/ml)	1.035	0.999–1.072	0.051			
TBil (μmol/L)	0.998	0.973–1.025	0.904			
DBil (μmol/L)	1.007	0.964–1.051	0.766			
**Medications**						
Aspirin (%)	0.778	0.505–1.199	0.256			
Statins (%)	1.112	0.705–1.753	0.648			
β-blocker (%)	0.567	0.320–1.004	0.052			
ACEI/ARB (%)	1.146	0.593–2.216	0.685			
CCB (%)	1.028	0.611–1.728	0.918			

### Predictors of Clinical Outcomes in Female Patients

**FIGURE 4:**
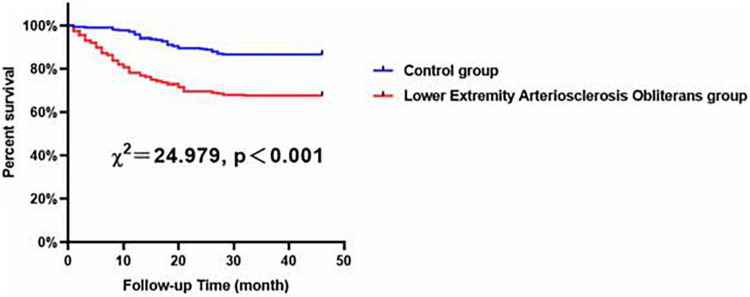
Major adverse cardiovascular and cerebrovascular events-free survival rates for female patients with LEASO group and control group during the follow-up period.

Compared with the female control patients, the female LEASO patients tended to be at a higher risk for the incidence of MACCEs during the follow-up period (χ^2^ = 24.979, *P* < 0.001, Figure 4).

Univariable Cox analysis demonstrated that hypertension, LEASO, WBC, neutrophils, lymphocytes, NLR, PLR, UA, glucose, Apo A1, fibrinogen, DBil, and the application of aspirin and β-blockers (all *P* < 0.05) were independent factors for MACCEs in all evaluated female patients in this study, as shown in Table 5. According to multivariate Cox analysis, independent influencing factors for the incidence of MACCEs in female patients included hypertension (HR: 2.010, 95% CI: 1.293–3.124, *P* = 0.002), LEASO (HR: 1.788, 95% CI: 1.110–2.880, *P* = 0.017), NLR (HR: 1.113, 95% CI: 1.041–1.190, *P* = 0.002), UA (HR: 1.002, 95% CI: 1.000–1.004, *P* = 0.049), and application of aspirin (HR: 0.472, 95% CI: 0.311–0.715, *P* < 0.001) or β-blockers (HR: 0.321, 95% CI: 0.176–0.586, *P* < 0.001) (Table 5).

**TABLE 5:** Predictors of the occurrence of MACCEs in female patients: results of univariate and multivariate Cox-regression analyses.

Indicators	Univariate			Multivariate		
	HR	95%CI	*p*	HR	95%CI	*p*
Age (years)	0.993	0.973–1.013	0.493			
Hypertension (%)	2.178	1.426–3.326	<0.001	2.010	1.293–3.124	0.002
Diabetes (%)	1.150	0.703–1.880	0.578			
LEASO (%)	2.845	1.849–4.377	<0.001	1.788	1.110–2.880	0.017
WBC (×10^9^/L)	1.117	1.070–1.166	<0.001	0.960	0.887–1.040	0.318
Neutrophil (×10^9^/L)	1.163	1.112–1.217	<0.001			
Lymphocyte (×10^9^/L)	0.541	0.375–0.779	<0.001			
NLR	1.121	1.088–1.155	<0.001	1.113	1.041–1.190	0.002
PLT (×10^9^/L)	0.998	0.995–1.001	0.212			
PLR	1.004	1.002–1.007	<0.001	0.998	0.995–1.002	0.424
Creatinine (μmol/L)	0.998	0.990–1.007	0.732			
UA (μmol/L)	1.002	1.001–1.004	0.005	1.002	1.000–1.004	0.049
Glucose (mmol/L)	1.147	1.071–1.230	<0.001	1.076	0.988–1.171	0.093
TG (mmol/L)	0.913	0.693–1.203	0.517			
TC (mmol/L)	0.991	0.788–1.248	0.942			
HDL-C (mmol/L)	1.042	0.568–1.910	0.894			
LDL-C (mmol/L)	1.060	0.812–1.384	0.668			
Apo A1 (g/L)	0.287	0.123–0.668	0.004	0.624	0.241–1.617	0.332
Apo B (g/L)	1.611	0.656–3.956	0.298			
Lp (a) (g/L)	0.999	0.999–1.000	0.439			
hs-CRP (mg/L)	1.002	0.994–1.010	0.594			
Fibrinogen (g/L)	1.384	1.201–1.595	<0.001	1.168	0.970–1.406	0.100
D-dimer (ug/ml)	1.061	0.992–1.136	0.086			
TBil (μmol/L)	1.014	0.988–1.041	0.290			
DBil (μmol/L)	1.058	1.014–1.103	0.009	1.026	0.961–1.096	0.441
**Medications**						
Aspirin (%)	0.607	0.407–0.903	0.014	0.472	0.311–0.715	<0.001
Statins (%)	0.793	0.527–1.195	0.268			
β-blocker (%)	0.397	0.221–0.712	0.002	0.321	0.176–0.586	<0.001
ACEI/ARB (%)	1.315	0.733–2.359	0.358			
CCB (%)	1.404	0.913–2.160	0.122			

### Sex Differences in the Characteristics and Prognosis of Lower Extremity Arteriosclerosis Obliterans Patients

**FIGURE 5:**
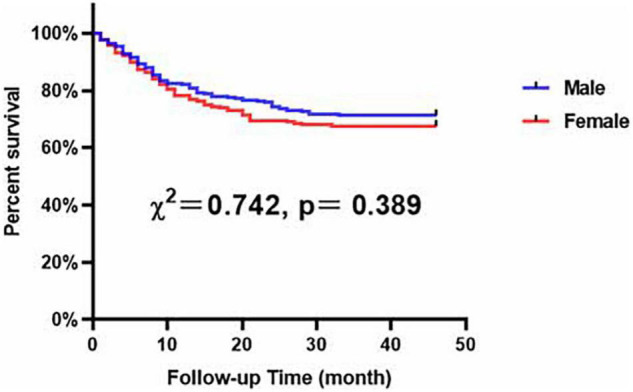
Major adverse cardiovascular and cerebrovascular events-free survival rates for male and female patients with LEASO during the follow-up period.

Our results demonstrated that, compared with male patients with LEASO, female patients with LEASO remained more likely to suffer from hypertension and had higher levels of HDL-C and Apo B and lower levels of UA (Table 6). In addition, Kaplan–Meier analysis indicated no significant difference in event-free survival rate between male and female LEASO patients (χ^2^ = 0.742, *P* = 0.389, Figure 5).

**TABLE 6:** Characteristics of male and female patients with LEASO.

	Male (*n* = 223)	Female (*n* = 207)	*t*/*Z*/χ ^2^	*p*
Age (years)	66.88 ± 11.56	66.19 ± 11.54	0.619	0.536
Hypertension (%)	100 (44.8)	116 (56.0)	5.382	0.020
Duration of Hypertension (years)	10.00 (6.25, 13.75)	10.00 (5.25, 15.00)	0.161	0.872
Diabetes (%)	43 (19.3)	44 (21.3)	0.259	0.611
Duration of Diabetes (years)	10.00 (6.00, 14.00)	10.00 (5.00, 14.50)	0.672	0.502
WBC (×10^9^/L)	7.89 ± 3.45	7.58 ± 3.99	0.723	0.470
Neutrophil (×10^9^/L)	5.45 ± 3.32	5.21 ± 3.62	0.727	0.468
Lymphocyte (×10^9^/L)	1.52 ± 0.54	1.55 ± 0.63	0.476	0.634
NLR	3.05 (1.94, 5.04)	2.63 (1.81, 4.47)	1.593	0.111
PLT (×10^9^/L)	219.01 ± 88.10	209.27 ± 80.67	1.193	0.234
PLR	139.07 (96.27, 191.71)	133.09 (102.38, 188.41)	0.454	0.650
Creatinine (μmol/L)	71.04 ± 12.12	71.79 ± 12.28	0.631	0.529
UA (μmol/L)	382.32 ± 120.26	353.34 ± 111.42	2.587	0.010
Glucose (mmol/L)	6.19 ± 2.69	6.23 ± 2.45	0.151	0.880
TG (mmol/L)	1.15 (0.87, 1.63)	1.23 (0.94, 1.75)	1.526	0.127
TC (mmol/L)	3.94 ± 1.01	4.12 ± 1.07	1.731	0.084
HDL-C (mmol/L)	1.04 ± 0.30	1.13 ± 0.32	2.879	0.004
LDL-C (mmol/L)	2.25 ± 0.85	2.30 ± 0.90	0.620	0.536
Apo A1 (g/L)	1.26 ± 0.23	1.28 ± 0.22	0.710	0.478
Apo B (g/L)	0.74 ± 0.22	0.79 ± 0.24	2.088	0.037
Lp (a) (g/L)	178.00 (73.00, 375.00)	205.00 (87.60, 461.00)	1.299	0.194
hs-CRP (mg/L)	1.42 (0.57, 5.37)	1.62 (0.59, 7.02)	1.043	0.297
Fibrinogen (g/L)	3.69 ± 1.41	3.54 ± 1.30	1.173	0.242
D-dimer (ug/ml)	0.65 (0.38, 1.37)	0.78 (0.39, 1.76)	1.123	0.261
TBil (μmol/L)	11.50 (8.80, 15.30)	12.50 (8.90, 16.50)	1.005	0.315
DBil (μmol/L)	4.00 (3.10, 5.60)	4.00 (3.00, 6.20)	0.145	0.885
**Medications**				
Aspirin (%)	141 (63.2)	121 (58.5)	1.028	0.311
Statins (%)	141 (63.2)	136 (65.7)	0.286	0.593
β-blocker (%)	50 (22.4)	56 (27.1)	1.240	0.266
ACEI/ARB (%)	20 (9.0)	25 (12.1)	1.107	0.293
CCB (%)	38 (17.0)	48 (23.2)	2.536	0.111

## Discussion

We have confirmed that LEASO can serve as a potential and powerful predictor for MACCEs. Moreover, subgroup analysis based on sex showed that LEASO also remained an independent predictor for the occurrence of MACCEs. These important observations show that our results support that LEASO is a robust predictor of the occurrence of MACCEs, irrespective of sex.

Cardiovascular disease is a complication of LEASO, which is explained by the theory of “panvascular disease,” which considers the vascular system as a whole (17). LEASO and cardiovascular diseases share a common atherosclerosis pathology, and both present the same risk factors (17). In other words, LEASO may be considered a poor prognostic predictor for the incidence of MACCEs. A retrospective cohort study including 1442 ACS patients showed that patients with PAD of the lower extremities carried a higher risk for cardiovascular disease (18). Another retrospective cohort study showed that lower extremity PAD patients with simultaneous CAD had a completely increased risk of all-cause mortality and MACCEs, which is in good agreement with our clinical result (19). Although there have been many similar studies regarding the potential association between LEASO and CAD in recent years, few studies have considered quantifying the potential impact and comparing important demographic characteristics, including risk factor exposure history and blood biochemical test data. Our study indicated that LEASO is an important prognostic factor for MACCEs, regardless of whether the influence of lipid profiles, inflammatory markers and other potentially prognostic confounders is considered. Therefore, our study provides more detailed data support for the concept of panvascular disease and demonstrates the importance of establishing an interdisciplinary center for panvascular disease management. In particular, whether the LEASO differs between females and males and whether these assessments provide parallel prognostic intelligence remain uncertain.

Inflammatory cytokines follow various stages of atherosclerosis, emphasizing the vital function of inflammation in the pathogenesis of atherosclerosis (20, 21). Recently, the popularity of atherosclerotic inflammation theory has mainly focused on emerging indicators such as NLR and PLR (22–25). The NLR, a novel meaningful and easily obtained inflammatory biomarker, serves not only as an independent predictor of carotid plaque vulnerability but also as a key predictor of future CV events and all-cause mortality (26, 27). Moreover, previous studies reported a close association between PLR and adverse outcomes (28, 29). Therefore, NLR, PLR and other inflammatory indicators, including white blood cells and hs-CRP, were included to further explore the relationship between inflammation and LEASO in our study. Our results showed that LEASO patients were more likely to have higher NLR, PLR, WBC, and neutrophil counts than the control group was, which implied that they remained in a higher inflammatory state. In light of this, inflammation is thought to play a vital role in the underlying mechanism between LEASO and poor outcome. However, whether inflammation acts as a bridge or only shares a common trigger with LEASO remains to be identified.

At the same time, we examined other measures of routine clinical test indicators, such as lipid profiles and coagulation function. Our results also found that patients with LEASO were inclined to have higher levels of TGs, TC, LDL-C, Apo B, and lipoprotein a and lower levels of HDL-C and Apo A1. The results of our study are consistent with previous results concerning atherosclerotic diseases, such as acute coronary syndromes and acute ischemic stroke (30, 31). Moreover, a previous study found that steady outpatients with PAD and higher levels of plasma fibrinogen had increased rates of equivalent ischemic events, which is consistent with findings in our study (32). Thus, our data also indicate that clinicians should attach importance to the routine examination results of LEASO patients, and timely intervention should be given to improve the prognosis of these patients.

Cardiovascular risk factors integrating sex-specific research have shown that although males and females share similar risk factors for CAD, certain risk factors are more potent in women (33). In particular, men remain more likely to suffer from ischemic heart disease, and women with coronary artery disease rarely present syndromes (34). Furthermore, compared to men, women more often experience less atherosclerotic plaque, manifested by chest pain and a lower risk of subsequent myocardial infarction (35). Our available data demonstrated that LEASO is an effective predictor in women as men, with a LEASO relative to a twofold increase in the risk of MACCEs. However, given sex-specific cardiovascular risk factor characterization, we found that female patients with LEASO tend to be more susceptible to hypertension, whereas male patients with LEASO are more at risk for higher Apo B and UA and lower HDL-C. Our studies were in the agreement with the previous studies indicated that the prevalence of hypertension is higher among post-menopausal women than among both premenopausal women and men (36–38). Additionally, our findings, together with previous observations that men have higher levels of UA (39) and hyperlipidemia (40), relative to women, which may be because of a high frequency of smoking, higher body mass index and other cardiovascular risk factors in men (40, 41). Thus, we should be cognizant of sex-specific cardiovascular risk factors in patients with LEASO. Further planning of effective preventive interventions for multiple cooccurring drivers may indicate poor clinical outcomes and provide patients with optimal clinical treatment decisions.

## Study Limitations

There are several limitations of our study. First, our diagnosis was based on the clinical record system, which means that our inclusion in the study was influenced by the experience of the clinician. Second, this study is retrospective; thus, our results may be subject to much bias (such as recall bias). Third, we eliminated many known confounding factors, but there is no guarantee about other unknown confounding factors. Fourth, we did not quantitatively assess atherosclerotic plaques in the lower extremities.

## Conclusion

This study indicates that LEASO tends to be a useful risk-stratified indicator for MACCEs in both male and female patients, regardless of sex. Where applicable, we highlight that attention should be given to patients with LEASO regardless of other risk factors and who should undergo comprehensive cardiovascular evaluation and intervention. Moreover, appropriate prevention programs should be tailored to different sex LEASO groups as well as different risk factors.

## Data Availability Statement

The datasets used and/or analyzed during this study are available from the corresponding author on reasonable request. Requests to access these datasets should be directed to LY, lileiyu@whu.edu.cn.

## Ethics Statement

Ethical approval was not provided for this study on human participants because this was a retrospective observational study, the Renmin Hospital of Wuhan University Ethics Committee granted an exemption from requiring ethics approval from eligible patients was waived. Written informed consent was not provided because this was a retrospective observational study, the Renmin Hospital of Wuhan University Ethics Committee granted an exemption from requiring informed consent from eligible patients was waived.

## Author Contributions

LY and HF made substantial contributions to conception and design, data acquisition, or data analysis and interpretation. JS, QD, JW, SD, HC, HZ, ZZ, FY, FG, CL, SX, LS, and YW drafted the manuscript or critically revised it for important intellectual content. LY gave final approval of the version to be published and agreed to be accountable for all aspects of the work in ensuring that questions related to the accuracy or integrity of the work were appropriately investigated and resolved. All authors contributed to the article and approved the submitted version.

## Conflict of Interest

The authors declare that the research was conducted in the absence of any commercial or financial relationships that could be construed as a potential conflict of interest.

## Publisher’s Note

All claims expressed in this article are solely those of the authors and do not necessarily represent those of their affiliated organizations, or those of the publisher, the editors and the reviewers. Any product that may be evaluated in this article, or claim that may be made by its manufacturer, is not guaranteed or endorsed by the publisher.
